# SOP: acute hyperkinetic movement disorders

**DOI:** 10.1186/s42466-023-00260-w

**Published:** 2023-07-27

**Authors:** Anna Sauerbier, Alexandra Gronostay, Haidar S. Dafsari

**Affiliations:** 1 Faculty of Medicine, Department of Neurology, University of Cologne, University Hospital Cologne, Cologne, Germany; 2 Department of Neurology, University Hospital Cologne, Kerpener Str. 62, 50937 Cologne, Germany

## Abstract

**Introduction:**

Movement disorders emergencies describe acute-onset neurological conditions in which a delay of recognition and treatment may cause severe morbidity and mortality of patients. Hyperkinetic movement disorders include tremor, chorea/ballism, dystonia, myoclonus, and tics. Here we present a standard operating procedure (SOP) for the diagnostic work-up and different treatment options depending on the phenomenology as well as the aetiology of underlying diseases.

**Comments:**

The recognition of the phenomenology is essential for the symptomatic therapy of the acute movement disorder and forms the basis for the choice of ancillary investigations to confirm the suspected underlying causes. Furthermore, we summarise diagnostic techniques, including blood and cerebrospinal fluid tests and neuroimaging, which provide rapid results and are useful for the indication of causal treatments of specific acute movement disorders.

**Conclusions:**

Despite their acute nature, most of these conditions can result in good clinical outcomes, if recognised early.

## Introduction

Conceptually, movement disorders emergencies describe acute-onset neurological conditions in which a delay of recognition and treatment may cause severe morbidity and mortality of patients. Hyperkinetic movement disorders are the most common emergencies of movement disorders and include [1–3]:

- **Tremor**
- **Chorea/ballism**
- **Dystonia**
- **Myoclonus**
- **Tics**

**Fig. 1:**
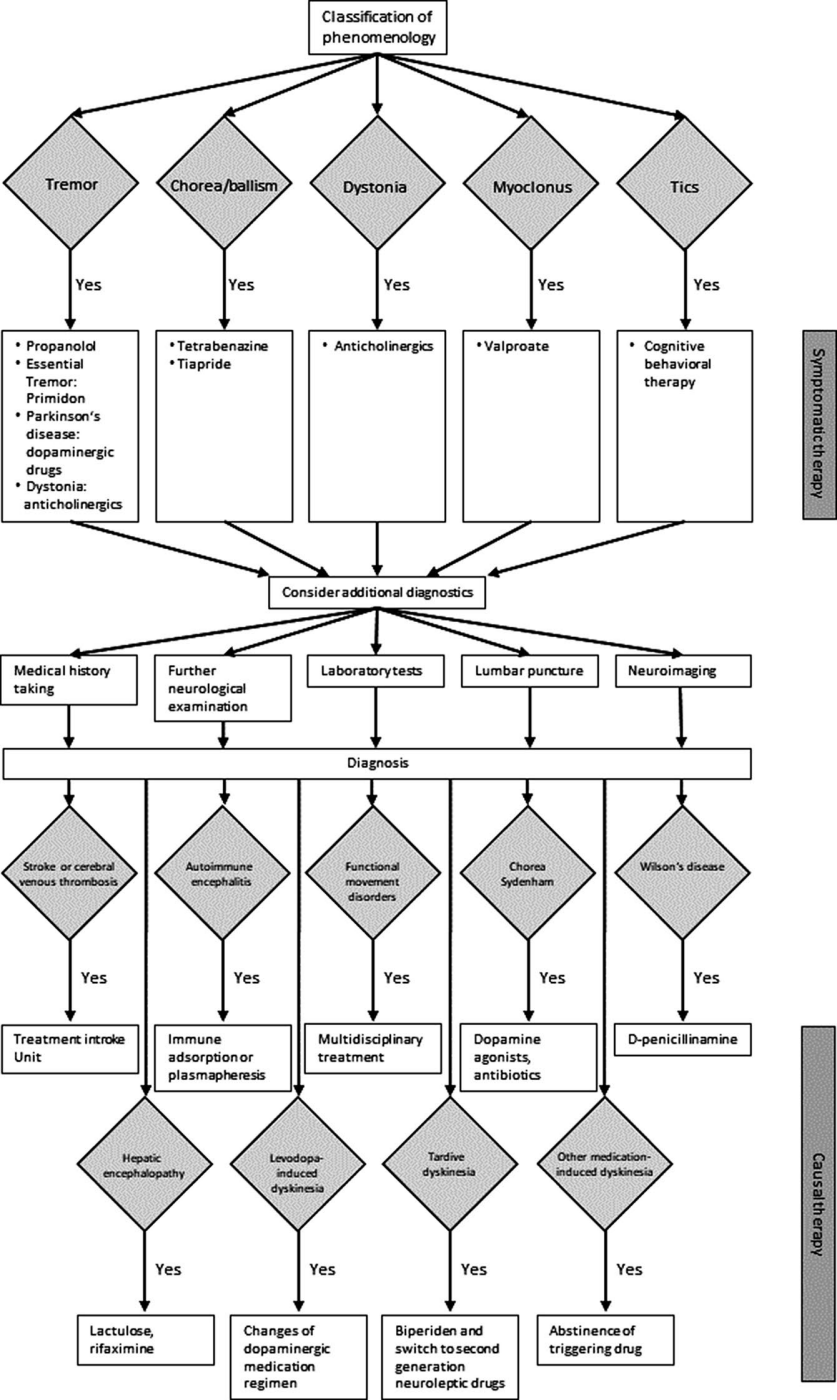
Flow chart SOP acute hyperkinetic movement disorders

Patients with acute onset and/or aggressive worsening of these hyperkinetic symptoms can present in emergency departments and intensive care units and require immediate neurological management [4]. Diagnostics and therapy are based on the classification of the hyperkinetic movement disorder phenomenology (see Fig. 1). Metabolic, drug-induced, and functional causes are relatively frequent, whereas genetic, autoimmune, and in industrial nations infectious causes are rare. Based on guidelines, current literature, and personal experience, we developed this standard operating procedure (SOP) on acute-onset hyperkinetic movement disorders focussing on their recognition and treatment during critical situations.

## Definitions

**Tremor** is a rhythmic and oscillating movement of one or more antagonistic groups of muscles.

**Chorea** is a non-suppressible, non-repetitive, continuously present sequence of one or more involuntary movements and **ballism** has the same characteristics but involves proximal muscles resulting in violent slingshot movements of the extremities. Hemichorea or hemiballism presents in in one side of the body.

**Dystonia** is a non-suppressible movement disorder, in which intermittent or continuous muscle contractions can lead to repetitive movements or abnormal posture.

**Myoclonus** is a sequence of non-suppressible, repetitive, often non-rhythmic, brief electric-shock-like jerky movements due to sudden contractions (positive myoclonus) or relaxation (negative myoclonus) of one or more muscles.

**Tics** are at least partially suppressible, short, regularly or irregularly repetitive movements which result in stereotypic, simple or complex movements or sounds uttered unintentionally. Performing a tic may provide relief to patients.

Furthermore, dyskinesia refer to involuntary movements presenting, e.g., as dystonia, chorea, or tics. Dyskinesia can present as part of several medical disorders with different underlying causes and their treatment is based on the specific aetiology (see causal therapy section).

## Diagnostics

Medical history taking: Current and previous drugs and previous illnesses, including psychiatric diseases in, e.g., in suspected tardive or other drug-associated dyskinesia? Cardiovascular risk factors in stroke? Infections in chorea Sydenham? Headache in cerebral venous thrombosis and stroke?

Neurological examination: Phenomenology of acute movement disorder? Accompanying neurological deficits or psychiatric symptoms? The diagnosis of functional hyperkinetic movement disorders was traditionally based on the absence of signs of organic diseases and has evolved more recently. The neurological examination should focus on whether specific positive clinical signs are present: motor inconsistencies, resolvement with distraction and/or entrainment, or momentary fluctuations, often in response to suggestion and/or placebo?

Laboratory tests: electrolytes and blood sugar (point-of-care test), and emergency laboratory tests including parameters for inflammation, infection, and tissue damage. Laboratory tests can be extended specifically: medication levels in suspected intoxication, ammonium in hepatic encephalopathy, peripheral blood smear in suspected neuroacanthocytosis. Specific antibodies can be tested, e.g. anti-streptolysin O titres in suspected chorea Sydenham, anti-double stranded DNA antibodies in systemic lupus erythematodes, anti-neuropil, anti-glutamic acid decarboxylase (GAD)-antibodies, and onconeuronal antibodies binding to nuclear or cytoplasmatic proteins or neuronal surface antibodies in suspected autoimmune encephalitis, and ceruloplasmin, serum and urine copper in Wilson’s disease.

Lumbar puncture can be conducted in suspected cases with encephalitis and aforementioned antibodies can be tested.

Neuroimaging: Preferentially MRI imaging with stroke protocol (DWI, FLAIR, SWI/T2*, TOF, and individual extensions) as ischaemic and haemorrhagic strokes can cause acute-onset movement disorders. If results are negative or unclear, add T1 contrast agent MRI, as structural and infectious lesions can also cause acute-onset movement disorders.

## Therapy

### Symptomatic therapy based on phenomenology of the acute hyperkinetic movement disorder

For tremor: Propanolol 30–320 mg/d, primidone 25–250 mg/d (essential tremor), dopaminergic medication (tremor in Parkinson’s disease and atypical parkinsonism), trihexyphenidyl 3–15 mg/d in dystonic tremor [5].

For dystonia: trihexyphenidyl (adults 1–16 mg/d, children and young adults tolerate higher daily dosages up to 100 mg/d). A medication-refractory dystonic crisis can be successfully treated with botulinum injections or deep brain stimulation in the internal globus pallidus [6].

For chorea: Dopamine-depleting drugs (tetrabenazine 25–200 mg/d) or dopamine receptor antagonists (tiapride 150–600 mg/d, the effect of treatment may not be apparent until a period of 6 weeks of treatment) [6].

For myoclonus: Valproate as first-line (stepwise initiation, effective medication level 50–100 mg/l), possibly benzodiazepine [7, 8].

For tics: Cognitive behavioural therapy as first-line therapy, possibly tiapride as second-line.

### Causal therapy based on aetiology of the hyperkinetic movement disorder

For ischaemic or haemorrhagic strokes and cerebral venous thrombosis: treatment in a stroke unit [9].

For autoimmune encephalitis: steroid pulse therapy, intravenous immunoglobines (2 g/kg body weight administered in divided doses over 2 to 5 consecutive days), immune adsorption or plasmapharesesis [6] (also see “SOP: antibody-associated autoimmune encephalitis” 10).

For functional movement disorders: multidisciplinary treatment including physiotherapy, psychotherapy and/or psychosomatic therapy [11].

For chorea Sydenham: dopamine agonists, consider antibiotic therapy and valproate [6, 12].

For Wilson’s disease: D-penicillinamine (10–20 mg/kg KG) [6].

For hepatic encephalopathy: lactulose (0.5-2 packages/d), rifaximine (1100 mg/d) [13].

For levodopa-induced dyskinesia including diphasic dyskinesia (typically presenting as dystonic or ballistic movements in the lower extremities), peak-dose dyskinesia (typically presenting as choreatic movements in the upper limbs), or off-dystonia (typically presenting as end-of-dose or early-morning off-dystonia with painful cramps in the foot): changes of dopaminergic medication regimen to avoid levodopa plasma levels, which trigger these dyskinesia [14]. Acute onset or worsening of peak-dose dyskinesia can be treated with amantadine (starting dose 100 mg/d) but concomitant hallucinations have to be considered as these might worsen under amantadine treatment [15]. Consider clozapine for concomitant hallucinations and other neuropsychiatric symptoms [16].

For tardive dyskinesia: early recognition is crucial. Biperiden for early tardive dyskinesia (2.5-5 mg i.v., monitoring ECG) and switch to second-generation neuroleptic drugs [17]. Chronic tardive dyskinesia can result from long-term neuroleptic treatment for psychiatric diseases and its treatment is challenging. Strategies include a discontinuation or switch of the psychiatric medication, which should be closely coordinated with psychiatrists.

Other medication-induced dyskinesia triggered by psychotropic medication, such as metamphetamine or cocaine (typically resulting in choreatic movements): the mainstay of treatment is abstinence [18].

## Conclusion

For clinicians encountering patients with acute hyperkinetic movement disorders, the recognition of the phenomenology is essential. The specific therapy depends on the phenomenology as well as the aetiology of underlying causes. Despite their acute nature, most of these conditions can result in good clinical outcomes [19]. Therefore, we emphasize the need to develop standard operating procedures and clinical pathways enabling a more prompt and accurate recognition and treatment of acute hyperkinetic disorders.
